# An Optimized Protocol for DNA Extraction from Wheat Seeds and Loop-Mediated Isothermal Amplification (LAMP) to Detect *Fusarium graminearum* Contamination of Wheat Grain

**DOI:** 10.3390/ijms12063459

**Published:** 2011-06-01

**Authors:** Kamel Abd-Elsalam, Ali Bahkali, Mohamed Moslem, Osama E. Amin, Ludwig Niessen

**Affiliations:** 1 King Saud University, College of Science, Botany and Microbiology Department, P.O. Box 2455, Riyadh 1145, Saudi Arabia; E-Mails: abhakali@ksu.edu.sa (A.B.); mbmoslem@ksu.edu.sa (M.M.); osamaemam@gmail.com (O.A.); 2 Center of Excellence in Biotechnology Research, King Saud University, P.O. Box 2455, Riyadh 11451, Saudi Arabia; 3 Plant Pathology Research Institute, Agricultural Research Center, Giza 12655, Egypt; 4 Technische Universität München, Lehrstuhl für Technische Mikrobiologie, Weihenstephaner Steig 16, D-85350 Freising, Germany; E-Mail: niessen@wzw.tum.de

**Keywords:** DNA isolation, loop-mediated isothermal amplification, LAMP, PCR, seed-borne fungi

## Abstract

A simple, rapid, and efficient method for isolating genomic DNA from germinated seeds of wheat that is free from polysaccharides and polyphenols is reported. DNA was extracted, treated with RNase, measured and tested for completeness using agarose gel electrophoresis. DNA purification from wheat grains yielded abundant, amplifiable DNA with yields typically between 100 and 200 ng DNA/mg. The effectiveness and reliability of the method was tested by assessing quantity and quality of the isolated DNA using three PCR-based markers. Inter-simple sequence repeats (ISSRs) were used to assess the genetic diversity between different wheat varieties. Specific PCR primer pair Tox5-1/Tox5-2 and a loop-mediated isothermal amplification (LAMP) procedure were used to detect genomic DNA of *Fusarium graminearum* in contaminated wheat seeds. In this method there is no need to use liquid nitrogen for crushing germinated seedlings. The protocol takes approximately one hour to prepare high quality DNA. In combination with the LAMP assay it is a fast and cost-effective alternative to traditional diagnostic methods for the early detection of toxigenic fusaria in cereals.

## 1. Introduction

It is generally quite difficult to extract and purify high-quality DNA from cereals because of the occurrence of polysaccharides, proteins, and DNA polymerase inhibitors such like tannins, alkaloids, and polyphenols in the extracts. The presence of these compounds reduces the quality and quantity of DNA which often make the sample non-amplifiable [1]. Polysaccharides are universal contaminants in plant extracts and can make DNA pellets slimy and difficult to handle. Particular problematic contaminants are those of an anionic nature which can be inhibitory to additional enzymatic analysis of the DNA, *i.e.*, restriction analysis [2]. DNA extraction performed on seeds has been previously described using halves of single dry seeds of wheat, barley, and rice [3] as well as seeds of other species [4] in order to perform RFLP and PCR based analysis of plant genotypes and genetic variation studies. Early diagnosis of *Fusarium* species is necessary for successful management of plant disease and subsequent prevention of toxins entering the food chain [5]. Several PCR assays have been developed to detect genes involved in the biosynthesis of mycotoxins [6]. Application of such assays in quality control may allow early detection of mycotoxin producing *Fusarium* spp. within a food or feed product [7]. Correlations between a positive detection result and the presence of trichothecene mycotoxins have been found [8]. Recently, a new nucleic acid amplification technique, loop-mediated isothermal amplification (LAMP) has been described as an easy and rapid diagnostic tool for the early detection of microbes [9]. LAMP assays have been developed for rapid detection of fungi in clinical samples [9–14], in plants [15,16] and during the process of brewing [17]. Loop-mediated isothermal amplification of DNA is a simple and rapid procedure for the specific detection of genomic DNA using a set of six oligonucleotide primers with eight binding sites hybridizing specifically to diverse areas of a target gene, and a thermophilic DNA polymerase from *Geobacillus stearothermophilus* for DNA amplification [18]. Recently, Niessen and Vogel [19] published a set of LAMP primers for the specific detection of *Fusarium graminearum* DNA which was based on the genomic DNA sequence of the *gaoA* gene. DNA isolation from cereal grains can be a very time-consuming and difficult procedure. However, in order to provide quality DNA for LAMP analysis, there is need for a rapid and easy-to-use low-cost protocol that can be adapted to the extraction and purification of DNA from cereal grains. The objectives of the present study were to develop a rapid and simple method for the direct extraction and purification of DNA from germinated wheat seeds. As a result, we describe the improvement of a single-seed DNA extraction protocol from germinated wheat seeds and demonstrate its usefulness to provide DNA template for the *F. graminearum* specific loop-mediated isothermal amplification of the *gaoA* target gene fragment as well as for the use with the group specific PCR primer pair Tox5-1/Tox5-2 [20] and with Inter-simple sequence repeat (ISSRs) DNA markers [21].

## 2. Materials and Methods

### 2.1. Plant Material

To investigate the genetic diversity of some wheat varieties from Saudi Arabia and Egypt using ISSR markers, nine wheat varieties (Giza 168, West Bred, Yecora rojo, Soundi 1, Soundi 2, Hab Ahmar and Lughaimi, Sama, and Sakha 61) were obtained from Ministry of Agriculture, Saudi Arabia and from Agricultural research Center, Giza, Egypt.

### 2.2. Germination of Seeds

Single seeds were germinated in a 24-well cell culture cluster (Costar Corporation, Cambridge, MA, USA) containing filter paper (2 cm), one seed per well and 1.5 mL sterilized water. Plates were placed in the dark at 27 ± 2 °C for 5 days.

### 2.3. Wheat Seedling Inoculation

**Figure 1:**
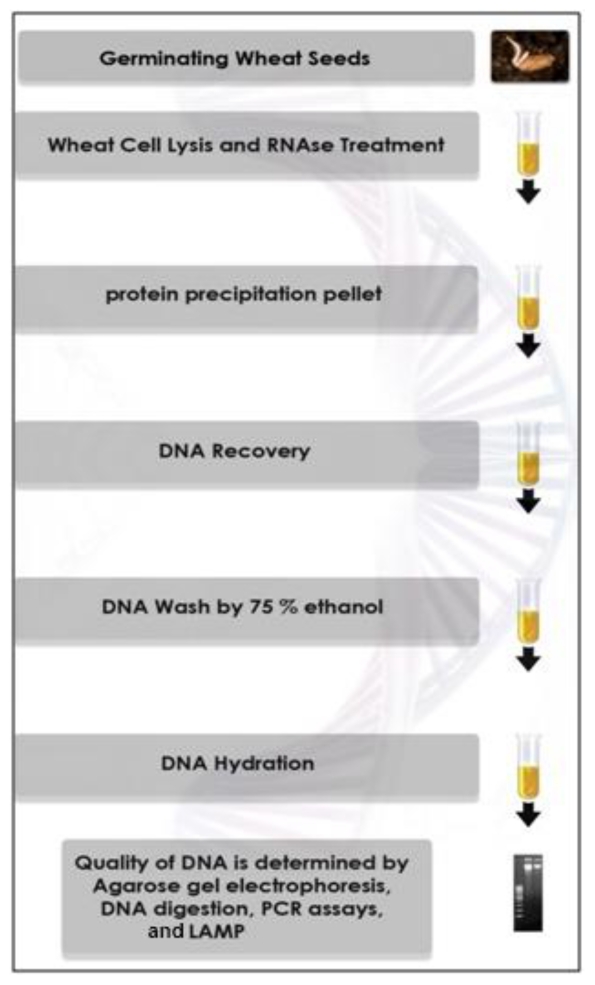
Flow diagram of the current method for DNA isolation from germinated wheat seeds.

Four strains of *F. graminearum* were inoculated on PDA slants and incubated for 2–4 weeks and spores were harvested as previously reported by Hilton *et al.* [22]. Wheat seedlings were inoculated by placing 10 μL of *F. graminearum* conidial suspension (containing 10^6^ conidia) on each emerging seedling leaf. Two leaves per seedling were inoculated. Control plants were treated with 10 μL of sterile distilled water. Inoculated plants were incubated in a moist chamber for 72 h and returned to the growth chamber at 21 °C (day)/16 °C (night) temperatures with day/night regime of 16 h/8 h. Total DNA of infested wheat samples was purified immediately after incubation as illustrated below (see Figure 1).

### 2.4. Fungal Cultures

Fungal isolates used in the current study are listed in Table 1. Fungal cultures were maintained at 10 °C on synthetic nutrient agar (SNA) medium [23] (0.5 g/L MgSO_4_·7 H_2_O, 1 g/L KNO_3_ (Roth, Karlsruhe, Germany), 0.2 g/L sucrose, 0.2 g/L glucose, 0.5 g/L KCl, 1 g/L KH_2_PO_4_ and 15 g/L agar (Merck, Darmstadt, Germany).

### 2.5. DNA Extraction

**Figure 2:**
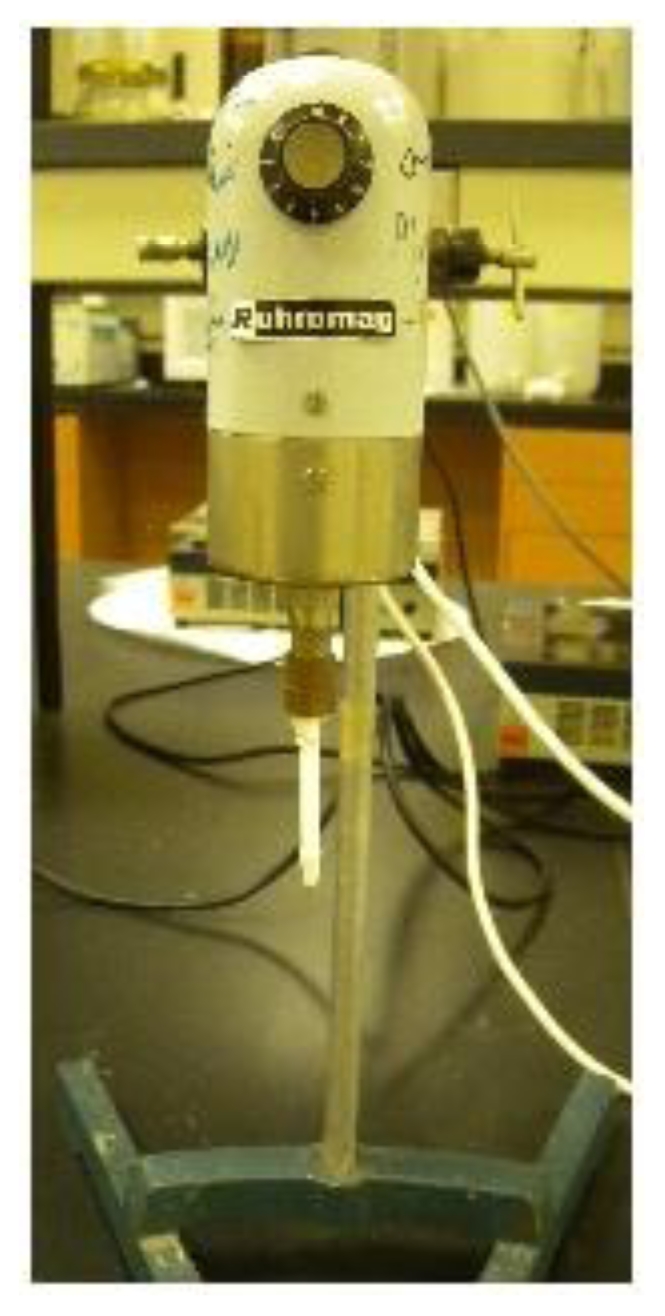
Electric homogenizer model R10 (Retsch, Haan, Germany).

For extraction of DNA from fungal mycelia, cultures were grown on disposable polystyrene Petri dishes (4 cm) which were filled with 1800 μL SNA. After solidification a layer of liquid medium (1400 μL Potato Dextrose Broth (PDB, 24 g/L; Scharlau, Barcelona, Spain) was added [24]. The fungal isolates were transferred by inoculating a small mycelial disk from the edge of an actively growing colony onto the prepared Petri dishes that were subsequently incubated for 2–3 days at 28 °C. The mycelium grows fast and can easily be scraped from duplex media. Prior to DNA extraction, mycelia were lifted from the solid medium using sterilized pipette tips and transferred into sterile 1.5 mL microfuge tubes. Alternatively, a clean and sterile spatula was used to scrape myclia from the surface of the agar media before transfer. Germinated wheat seeds were transferred individually to sterile 1.5 mL microfuge tubes for DNA extraction. A hand-operated as well as an electric grinder model R10 (Retsch, Haan, Germany) (Figure 2) was used to homogenize 100 mg of fresh mycelia or one germinated wheat seed (about 100 mg) per 1.5 mL microfuge tube.

To prevent cross-contamination of DNA, the pestle tip was sanitized between isolations by dipping in 70% ethanol and the homogenizer was turned on for 3 s and subsequently the pestle tip was rinsed in sterile distilled water and dried using sterilized filter paper [24]. The primary mechanical breakdown of germinated wheat seeds was carried out with DNA extraction buffer (300 μL) using a hand operated homogenizer instead of liquid nitrogen. Another three hundred micro liter pre-warmed (at 65 °C) DNA extraction buffer (200 mM Tris-HCl, pH 8.5, 250 mM NaCl, 25 mM EDTA, 0.5% SDS, 1% PVP) was added to the homogenized samples. Six micro liters of RNaseA (20 μg/mL water) were added and the microfuge tubes were incubated at 65 °C in a water bath for 15 min with irregular shaking and swirling every 5 min. For protein precipitation, 160 μL of 3 M sodium acetate (pH 5.3) were added and mixed carefully by inverting the tube. The mixture was centrifuged at 15,000× g for 8 min at room temperature. The supernatant was carefully transferred to a new tube and was precipitated with one equal volume of cold isopropanol at 4 °C, and thoroughly mixed to produce filamentous DNA followed by 10 min incubation at room temperature. Genomic DNA was collected by centrifugation at 8900× g for 10 min. The resulting pellet was washed twice with 75% ethanol at ambient temperature and subseqently centrifuged at 5700× g for 2 min. The pellet obtained was dried under vacuum using a Vacufuge Concentrator 5301 (Eppendorf, Hamburg, Germamy) at 37 °C for 5 min with <20 mbar pressure. In the final step, DNA was eluted in 100 μL of sterile deionized water and kept at −20 °C until used as template for PCR or LAMP amplification. The extraction protocol is sketched schematically in Figure 1 (see above).

The isolated DNA was measured by using the Nano-Drop (ND-1000) spectrophotometer (NanoDrop Technologies, Welmington, USA). DNA concentrations were confirmed using agarose gel electrophoresis. Ten microliters of purified DNA from the proposed procedure was run on a 1.5% (w/v) agarose gel containing 0.1 μg/mL of ethidium bromide. DNA was visualized using the Biorad Gel Doc System (Biorad, NSW, Australia).

### 2.6. Inter-Simple Sequence Repeats (ISSRs)

In this study, primer ISSR W8 containing eight CT-repeats was used for estimating genetic polymorphism of wheat varieties. PCR amplification was performed in a Techne Thermal Cycler-TC-3000 (Techne, Cambridge, UK). Polymerase chain reactions were performed using 50 ng of template DNA in 25 μL reaction volume using 0.2 unit of *taq* DNA polymerase (JenaBoscience, Jena, Germany) with the included reaction buffer containing (20 mM Tris-HCl, 10 mM (NH_4_)_2_SO_4_, 10 mM KCl, 2 mM MgSO_4_, 0.1% Triton X-100, pH 8.8), 20 pmol of ISSR W8 primer (see Table 2), 0.2 mmol/L of each dNTP. ISSR PCR was carried out in a final volume of 25 μL according to the following thermal cycling protocol: initial denaturation at 94 °C for 3 min; followed by 35 cycles of denaturating at 94 °C for 30 s, annealing at 45 °C for 30 s, elongation at 72 °C for 30 s, followed by a final extension step at 72 °C for 5 min.

### 2.7. Trichodiene Synthase (Tri5) Gene Assay

The PCR primers Tox5-1 and Tox5-2 were used to amplify a 658 bp fragment from the trichodiene synthase gene *tri5* of trichothecene producing *Fusarium* species [20]. The PCR mixture used was as described earlier and the PCR conditions were: initial denaturation at 94 °C for 3 min; followed by 35 cycles of denaturating at 94 °C for 30 s, annealing at 63 °C for 30 s, elongation at 72 °C for 1 min, followed by a final extension step at 72 °C for 7 min.

### 2.8. LAMP Reaction

Loop-mediated isothermal amplification (LAMP) of *F. graminearum* DNA was accomplished using the protocol described by Tomita *et al*. [25] with slight modifications. Six primers as described in Niessen and Vogel [19] were used in this study with nucleic acid sequences and concentrations as shown in Table 2. The master mix was prepared containing per 25 μL reaction: 2.5 μL 10 × LAMP buffer (200 mM Tris-HCl, 100 mM KCl, 100 mM (NH_4_)_2_SO_4_, 80 mM MgSO_4_, 1% (w/v) Triton X-100, pH 8.8, all chemicals from Sigma-Aldrich, Taufkirchen, Germany), 3.5 μL dNTP mix (10 mM each, MP Biomedicals, Heidelberg, Germany), 2.6 μL primer mix (1.6 mM FIP-gaoA ID4, 1.6 mM BIP-gaoA ID4, 0.2 mM F3-gaoA ID4, 0.2 mM B3-gaoA ID4, 0.8 mM LoopF-gaoA ID4, 0.8 mM LoopB-gaoA ID4, Eurofins MWG Operon, Ebersberg, Germany), 8 U (1.0 μL) Bst DNA polymerase, large fragment (8000 U/mL, New England Biolabs, Frankfurt, Germany), 1 μL calcein reagent (see below). Sterile deionized water was added to result in a 25 μL total reaction volume, including template DNA. 2 μL of template DNA solution were added per reaction. The calcein reagent was prepared by mixing a 2 × LAMP buffer (see above) in which MgSO_4_ was substituted by 25 mM MnCl_2_ with calcein (Sigma-Aldrich, Taufkirchen, Germany) to result in a 2.5 mM solution. This solution was diluted 1:1 with with 80% glycerol and filter sterilized through a 0.2 μm filter cartridge (Sartorius, Göttingen, Germany). Aliquots of the calcein reagent were stored at −20 °C. Master mixes were distributed into 200 μL PCR tubes (Multiply μStrip Pro 8-strip, Sarstedt, Nümbrecht, Germany) and sample DNA was added. Water was added instead of DNA in negative controls. Amplificaton of DNA during LAMP was indicated by a bright green fluorescence under UV_366nm_ light visible in the positive samples as compared to negative samples and the negative control with no fluorescence. Direct visualization of amplified product was achieved by separation of the LAMP reactions on 1.5% agarose gels following incubation. Gels were run at 60 V, 55 mA for 45 min and stained with ethidium bromide solution (1 g/mL). PCR amplicons were documented using a Biorad Gel Doc System (Biorad, NSW, Australia).

## 3. Results

**Figure 3:**
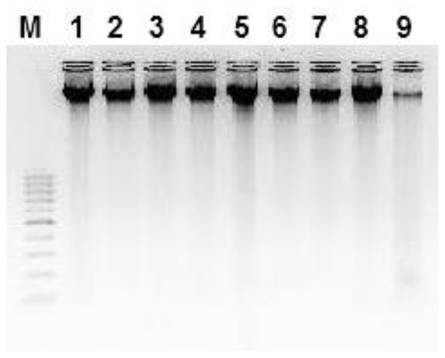
Agarose gel-electrophoresis of 100 ng representative wheat DNA extracted from germinated wheat seeds of different varieties. Lanes: M, 100 bp DNA marker, Lane 1, Giza 168; Lane 2, West Bred; Lane 3, Yecora rojo; Lane 4, Soundi 1; Lane 5, Soundi 2; Lane 6, Hab Ahmar; Lane 7, Lughaimi; Lane 8, Sama; and Lane 9, Sakha 10.

The DNA isolated was mainly high molecular DNA with only a very small proportion of lower molecular DNA (see Figure 3). It had minimal contamination by polysaccharides and phenolic compounds as was confirmed by good digestibility with restriction endonucleases as well as by a 260/280 nm ration around 1.8 indicating high purity of DNA and absence of proteins and phenols (data not shown). RNase treatment was sufficient for degrading RNA. Treatment with RNase A can be done either after the nucleic acids have been isolated or by inclusion in the extraction buffer [26]. The resulting DNA pellets were all colorless and dissolved easily in water. DNA yields ranged from 100 to 200 ng per 100 mg of homogenized material which is supposed to be sufficient material to conduct 200 PCRs (Figure 3).

**Figure 4:**
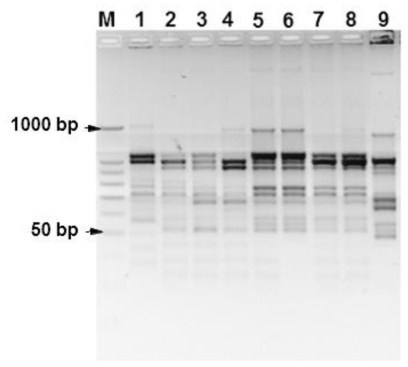
Random amplification of SSR markers from genomic DNA extracted from single seeds of different wheat varieties using the ISSR W8 primer. M, Bench Top PCR markers (Promega). Lane 1, Giza 168; Lane 2, West Bred; Lane 3, Yecora rojo; Lane 4, Soundi 1; Lane 5, Soundi 2; Lane 6, Hab Ahmar; Lane 7, Lughaimi; Lane 8, Sama; and Lane 9, Sakha 10.

In order to facilitate the efficiency and reliability of the DNA extraction method and the quality of the extracted DNA the seed DNA of wheat varieties was amplified using primer ISSR W8. It was confirmed that 21 ISSR fragments with the expected range of length between 50–2000 bp were amplified. Among these, 5 ISSR fragments were monomorphic in the tested varieties in the range from 90–900 bp (Figure 4). The percentage of polymorphic bands was 42% in the tested varieties. The ISSR W8 primer clearly differentiated between Egyptian (Giza 168 and Sakha 10) and Saudi Arabian wheat varieties.

Quality of the extracted fungal DNA was tested by using preparations from isolates of *F. cerealis*, *F. chlamydosporum*, *F. concolor*, *F. culmorum*, *F. graminearum*, *F. langsethiae*, *F. incarnatum*, *F. proliferatum* and *F. verticillioides* to test for amplifiability of a 658-bp fragment from the trichodiene synthase (*tri5*) gene [20]. Table 1 shows the strains used and the results obtained. Detection of a 658 pb product occurred in the species which have been described to be producers of trichothecene mycotoxins in the literature.

**Figure 5:**
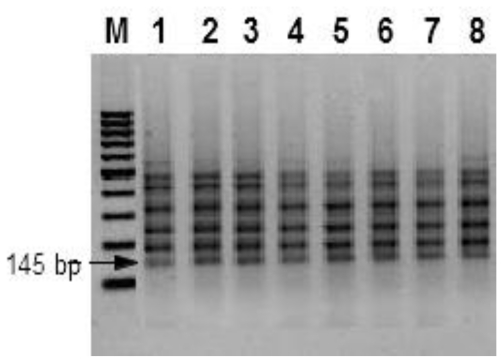
Agarose gel electrophoresis of loop-mediated isothermal amplification products amplified from genomic DNA extacted during the current study. 1 = *F. graminearum* PPathRI 0555, 2 = *F. graminearum* DSMZ 1096, 3 = *F. graminearum* DSMZ 893, 4 = *F. graminearum* DSMZ 4527, 5 = *F. culmorum* DSMZ 1094, 6–8 = DNA from wheat seedling after artificial inoculation with *F. graminearum* DSMZ 893.

LAMP reactions were accomplished using the primer set given in Table 1. The primers were published previously [19]. They were selected to identify six different regions on the *gaoA* gene of *F. graminearum*. A pair of loop primers was used in order to accelerate DNA amplification during LAMP. Detection of DNA amplification during LAMP under the conditions chosen was done routinely using the indirect calcein fluorescence reaction described by Tomita *et al.* [25]. In order to demonstrate congruence of the results obtained with calcein indirect fluorescence, LAMP reactions were separated on an agarose gel and stained with ethidium bromide. The size of the LAMP reaction products ranged from 145 bp up to the loading well on the agarose gel, indicating specific amplification of the target sequence (Figure 5). Visualization of DNA amplification during LAMP was achieved by inspection of the reaction vessels under UV_366nm_ trans-illumination after adding manganese quenched calcein to the master mix.

**Figure 6:**
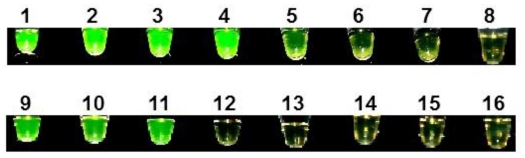
Visual appearance of LAMP-PCR products by using primer set gaoA ID4 and indirect calcein fluorescence detection under UV366 nm light. The reactions were incubated in a water bath for 60 min at 63 °C. 1 = *F. graminearum* PPathRI 0555, 2 = *F. graminearum* DSMZ 1096, 3 = *F. graminearum* DSMZ 893, 4 = *F. graminearum* DSMZ 4527, 5 = *F. culmorum* DSMZ 1094, 6 = *F. avenaceum* TMW 4.0140, 7 = *F. cerealis* DSMZ 8704, 8 = *F. chlamydosporum* DSMZ 62169, 9–11 = DNA from infested wheat seedling with *F. graminearum*, 12 = *F. verticillioides* DSMZ 62452, 13 = *F. oxysporum* PPathRI 0003, 14 = *F. oxysporum* f. sp. *vasinfectum* PPathRI 0421, 15 = *Alternaria alternata,* PPathRI 0500, 16 = *Aspergillus flavus* PPathRI 0111.

As shown in Figure 6, positive reactions showed brilliant green fluorescence in addition to turning turbid while negative reactions showed no fluorescence and no turbidity. Inspection of the reaction vessels under daylight showed that positive reactions turned from light orange to greenish-yellow with turbidity whereas negative reactions remained light orange and stayed clear. Positive results were obtained for the four *F. graminearum* isolates tested, while all reactions with *Fusarium* spp. or other fungal species included for comparison (Table 2) were negative with a response identical to the negative control to which no DNA was added. *F. culmorum* showed an intermediate reaction with a slight fluorescence in the LAMP reaction (see Figure 6, Sample 5).

Electrophoresis of the corresponding reaction showed that DNA was amplified under the reaction conditions in this sample (see Figure 5, lane 5). Intensity of the fluorescence signals obtained was independent from DNA concentrations in positive samples as long as their DNA concentrations were above the limit of detection. When DNA was extracted according to the new protocol from wheat seeds inoculated with *Fusarium* species, a fluorescent signal was produced only in samples infected with *F. graminearum* strains.(see Figure 6, Samples 9–11) indicating the effectiveness of the extraction method for the analysis of infected samples using species specific LAMP.

## 4. Discussion

The contamination of cereals by *Fusarium* species is a universal problem, and reliable and sensitive detection methods are needed. In order to use diagnostic PCR, rapid methods for isolation of *Fusarium* DNA were previously developed [27,28]. The major objective of the current study was to further optimize DNA extraction thus to allow constant quality of extracts and high efficiency of amplification in LAMP and PCR based methods. Mechanical grinding of cells directly in the DNA isolation buffer for extraction of DNA turned out to be very simple and cost effective since it did not need the use of liquid nitrogen. This is especially advantageous when huge numbers of samples need to be tested. Zhang and Stewart [29] described a similar extraction protocol that uses an electric drill equipped with a bit to which a plastic tip was molded, which fit the bottom of a 1.5 mL micro tube for grinding cotton tissue. They used the macerated tissue for CTAB-based DNA extraction. During the SDS lysis phase, proteins and polysaccharides become trapped in large complexes that are coated with dodecyl sulfate. Additionally, the SDS might bind to proteins also in the purification step of the extraction, thus inhibiting degradation of the extracted DNA [30,31]. In the current method, contaminants were further removed by an isopropanol precipitation of DNA. Also, RNA contamination was fully removed by RNase A. The addition of high molar concentrations of NaCl in the extraction buffer increases the solubility of polysaccharides in ethanol, thus effectively inhibiting co-precipitation of the polysaccharides together with the DNA [32]. PVP in the extraction buffer could promote the quantity and purity of DNA. According to Zidani *et al.* [33], PVP promotes precipitation of phenolic compounds which often adhere to DNA forming stained extracts and interfering with DNA amplification reactions. During the current study the quality of DNA extracts was checked by determining the ratio of absorbance at A260/280. The ratio obtained varied from 1.6 to 1.8 indicating that the isolated DNA was free from protein contamination [34]. Suitability of the DNA extraction protocol was demonstrated by applying three different types of amplification technologies. Random amplification of inter-simple sequence repeats or micro satellites was achieved using a primer which hybridizes to simple sequence repeats (SSRs) thus allowing amplification of the spacer regions between two binding sites. Results showed that microsatellites were amplified with the genomic DNA extracted from single seeds of different wheat varieties. The method described is therefore suitable for routine application in high-throughput molecular genetic analyses, such as breeding programs and gene mapping [35]. Specific PCR amplification of a 658 bp portion of the *tri5* gene was done using primers Tox5-1 and Tox5-2 as reported by Niessen and Vogel [20]. Amplification resulted in a single band of 650 bp in agarose gels for isolates of *F. cerealis*, *F. chlamydosporum*, *F. concolor*, *F. culmorum*, *F. graminearum*, *F. langsethiae*, *F. incarnatum*, *F. proliferatum* and *F. verticillioides* studied. Except for the latter two, all species have been described as producers of thrichothecene mycotoxins in the literature and can therefore be supposed to have a homolog of the *tri5* gene. *F. proliferatum* and *F. verticillioides*, however, do not produce trichothecenes but are typical producers of fumonisins. Positive reaction of the DNA extracted from the two species with the *tri5* specific primers was unexpected and could be a result of DNA transfer from other preparations during extraction. It is therefore of extreme importance to thoroughly clean all equipment before starting the next sample preparation. LAMP using a primer set targeted to the *gaoA* gene was used for detecting *F. graminearum* contamination in wheat seeds as well as to identify pure cultures of the fungus. The assay developed by Niessen and Vogel [19] is cost-effective and suits high throughput detection of *F. graminearum* in wheat seeds. The LAMP assay is practically equipment-free, requiring only a device such as a water bath or simple heating block to maintain a constant temperature. It is therefore much less technically demanding as compared to PCR. Detectable copies of DNA were obtained after only 1 h of incubation under isothermal incubation at 63 °C, and visual evaluation of the outcome could be carried out using a simple UV lamp. A bright green fluorescence was easily detectable in samples in which DNA synthesis had taken place under the given conditions whereas negative samples did not fluoresce. The major advantage over other fluorescent stains, e.g., ethidium bromide [36], SYBR green, propidium iodide [37], Eva green [38,39] and the like which intercalate into the double stranded DNA structure, is that no inhibition of the amplification process occurs. The LAMP fluorescence detection reagent contains manganese loaded calcein which starts fluorescing after removal of the bivalent cation by complexation with pyrophosphate, a typical by product of DNA biosynthesis [19,25,40]. Thus no interaction between the stain and the process of DNA amplification occurs.

## 5. Conclusions

We describe a new and rapid protocol for DNA isolation from single wheat seeds and fungal mycelia which is fast, consistent and low-priced. The current method can be used for DNA extraction from developing (immature) seeds of cereals, such as barley, maize, sorghum, *etc.* The method can be used to prepare DNA from numerous wheat grains in short time for molecular analysis of plant varieties with ISSR based methods as well as specific amplification of DNA in PCR- and LAMP-based assays for the detection of toxigenic *Fusarium* spp. in cereals and pure cultures. The proposed method enables the extraction of DNA from a large number of samples rapidly and efficiently. It does not require expensive and environmentally hazardous reagents and equipment. It can therefore be performed even in low-technology laboratories for high throughput sample preparation suitable for various molecular analytical techniques.

## Figures and Tables

**Table 1:** List of fungal species and isolates used to evaluate primer specificity in the *tri5* gene specifc PCR and *F. graminearum* specific LAMP assays.

Fungal Species	Reference Strain	tri5 Gene	LAMP GaoA ID4
*F. avenaceum*	DSMZ 62785	−	−
*F. avenaceum*	TMW 4.0140	−	−
*F. cerealis*	DSMZ 8704	+	−
*F. chlamydosporum*	DSMZ 62169	+	−
*F. chlamydosporum*	PPathRI 0222	+	−
*F. concolor*	DSMZ 62179	+	−
*F. culmorum*	DSMZ 1094	+	−
*F. graminearum*	PPathRI 0555	+	+
*F. graminearum*	DSMZ 1096	+	+
*F. graminearum*	DSMZ 893	+	+
*F. graminearum*	TMW 4.0208	+	+
*F. langsethiae*	TMW 4.0072	+	−
*F. incarnatum*	DSMZ 62403	+	−
*F. proliferatum*	DSMZ 62376	+	−
*F. verticillioides*	DSMZ 62452	+	−
*F. oxysporum*	PPathRI 0003	−	−
*F. oxysporum* f. sp. *vasinfectum*	PPathRI 0421	−	−
*Alternaria alternata*	PPathRI 0500	−	−
*Aspergillus flavus*	PPathRI 0111	−	−
*Macrophomina phaseolina*	PPathRI 0342	−	−
*Rhizoctonia solani*	PPathRI 0471	−	−

TMW = Technische Mikrobiologie Weihenstephan, Freising, Germany; DSMZ = Deutsche Sammlung von Mikroorganismen und Zellkulturen, Darmstadt, Germany; PPathRI = Plant Pathology Research Institute, Giza, Egypt.

**Table 2:** Primers list used in the current study.

Primer Code	Primer Sequence	Concentrations
ISSR W8	5′-CTC TCT CTC TCT CTC TCT-3′	20 pmol
Tox5-1	5′-GCT GCT CAT CAC TTT GCT CAG-3′	20 pmol
Tox5-2	5′CTG ATC TGG TCA CGC TCA TC-3′	20 pmol
FIP-gaoA ID4	5′-CGC AAG TGA CGG CCC AGT TGC TTC GAG CCT CAG CAC CTA-3′	1.6 mM
BIP-gaoA ID4	5′-TGC AAC AAG GCC ATT GAT GGC CGT TGG CGC CAT AGA ATG T-3′	1.6 mM
F3-gaoA ID4	5′-AGG GAG TCT TCA GTT CCT GA-3′	0.2 mM
B3-gaoA ID4	5′-GTG AGG GGG CTT TGG ATC-3′	0.2 mM
LoopF-gaoA ID4	5′-GTT GCG AGA AAT GGC GCT TCC G-3′	0.8 mM
LoopB-gaoA ID4	5′-ACA AGG ATA CCT TTT GGC AC-3′	0.8 mM

All nucleotides were purchased from Eurofins MWG Operon, Ebersberg, Germany.
